# Domains of competence in midwifery students: a basis for developing a competence assessment tool for iranian undergraduate midwifery students

**DOI:** 10.1186/s12909-022-03759-z

**Published:** 2022-10-05

**Authors:** Firoozeh Firoozehchian, Armin Zareiyan, Mehrnaz Geranmayeh, Zahra Behboodi Moghadam

**Affiliations:** 1 Department of Reproductive Health and Midwifery, School of Nursing and Midwifery, Tehran University of Medical Sciences, Tehran, Iran; 2 Department of Public Health, Department of Health in Disaster & Emergencies, Nursing Faculty, Aja University of Medical Sciences, Tehran, Iran; 3 Department of Reproductive Health and Midwifery, Faculty of Nursing and Midwifery, Tehran University of Medical Sciences, Tehran, Iran

**Keywords:** Clinical competence, Midwifery students, Midwifery, Domains, Competence, Midwifery Education

## Abstract

**Background:**

Current study was conducted with the aim of explaining domains of clinical competence in undergraduate midwifery students so that it addresses the challenges in midwifery curriculum and improving clinical assessment methods in Iranian undergraduate midwifery students.

**Methods:**

Qualitative approach and conventional content analysis were used in the design of the present study. The research setting included midwifery and nursing schools and hospitals and health centers affiliated to Tehran and Guilan universities of medical sciences in Iran. The target population consisted of undergraduate midwifery students in the fourth to eighth semesters of school, midwives working in hospitals and health centers, midwifery faculty members, and obstetricians. The participants were selected through purposive maximum variation sampling, which continued until data saturation. After in-depth semi-structured interviews, the content of the interviews was analyzed according to the steps proposed by Zhang & Wildemuth.

**Results:**

Twenty-four people participated in this study, including seven midwifery students, seven midwives, nine midwifery and reproductive and sexual health faculty members, and one obstetrician. The participants were aged 20–56 years and their mean age was 39.75 years. Their level of education varied from midwifery student to PhD. The mean work experience of the participants was 13.62 years and the mean duration of the interviews was 48 min. The analysis of the data obtained from the experiences of the participants led to the formation of the four categories of ethical and professional function in midwifery, holistic midwifery care, effective interaction, and personal and professional development, along with ten subcategories.

**Conclusion:**

The findings of the present study showed that clinical competence in midwifery students involves different domains that correspond well overall to the general definitions of clinical competence in different sources. These findings can be used as a basis for the design and psychometric assessment of a clinical competence assessment tool for undergraduate midwifery students.

## Background

Midwifery education in Iran is performed at the Bachelor’s, Master’s, and Ph.D. levels. The Ministry of Health and Medical Education of Iran has designed midwifery curricula at all levels for universities in the country. Graduated midwives can work in hospitals, health centers, universities, or private offices depending on their level of education [1].

Undergraduate midwifery is a four-year program in Iran. A significant amount of time during this program (50%) is devoted to teaching a variety of clinical skills in different practices. After the first semester, students enter clinical settings in groups of four to eight under the supervision of clinical educators. Clinical educators are full-time or part-time instructors working in midwifery and nursing schools with a master’s degree or PhD in Reproductive and Sexual Health or a PhD in Midwifery and are *not* midwives working in hospitals or health centers. Childbirth services in maternity wards in Iran are mostly organized by the medical care model, and midwives work under the supervision of obstetricians [2].

According to a report by the WHO in 2014, investing in midwifery education with a focus on community-based services can be up to 16 times more profitable in terms of preventing deaths and avoiding the cost of cesarean section. The quality of the work of midwifery care providers can be improved with upgrading the quality of midwifery education. Although educational program in the most countries are appropriate and updated, universal gaps in educational structure systems and resources [3]. Following the protest of women in New Zealand to over-medicalization of childbirth, New Zealand midwives also demanded the independence of midwifery and return to their professional identity. The result of these efforts is now the successful model of midwifery education in New Zealand based on education, practice and independence. This model of midwifery education combines academic learning, practice, and it trains capable and self- confident midwives, who can work independently in clinical fields [4]. The knowledge and professional competence of midwives is necessary to establish a sense of trust between the woman and the midwife. Competence in providing efficient and effective care is one of the twelve areas identified in the concept of Respectful Maternal Care (RMC) [5]. It has been found that the women who receive midwifery care, compared to women who receive medical care, have expressed more satisfaction with their care and delivery experience [6].It seems that the reason for that issue is due maintaining the basic values of care by midwives and midwifery care is more aligned with the criteria of (RMC) [7, 8].

The midwifery profession requires knowledge, competence, self-confidence, and skills [9].

Competence means having sufficient information, psychomotor skills, communication skills, decision-making power, and attitude to perform certain tasks. The reproductive and sexual health core competencies in primary healthcare include having proper attitude, knowledge, and ethics, respecting human rights, ability to lead, manage, and conduct teamwork, ability to perform social activities, and ability to offer education, counseling, and clinical services [10]. Professional performance depends not only on the tasks that have to be performed but also on the needs, resources, healthcare structure, and social and economic conditions [11].

Midwives should also use their personality traits in clinical practice, such as empathy and intuition, to be able to interact with women in a personal and professional manner [12].

Midwife should possess enough skill and resources to respond different and personal need of women [13].In woman-centered care, the midwife tries to empower women, has a respectful partnership with them, supports them, does not judge them, and is honest and sympathetic. The midwife is a professional and at the same time sensitive and intimate that keep confidentiality and support family [14].

The International Confederation of Midwives (ICM) established the first set of core competences for basic midwifery practices in 2002, which was updated in 2010 and amended in 2013. These competences provide an important framework for midwifery educators and regulatory bodies around the world and represent the highest standard for midwifery education and clinical practice [15]. The updated ICM competences have been organized in four interrelated categories, are written as comprehensive sentences, and reflect the midwifery care model [16]. Educational institutions are currently inclined toward competence-based education and have realized the significance of promoting graduates’ readiness for practice and ensuring that they are capable of providing safe care; therefore, improving students’ learning and pointing them to the right direction have become more important than ever[17]. Today, educators are increasingly being required to accurately measure and report on their students’ competences, while the lack of valid and reliable criteria for evaluating these competences has been an impediment to valid assessments [18, 19]. Deciding about whether or not students have achieved the required learning outcomes is based on their actual performance. Therefore, competence assessment in practical environments requires competence-based assessment tools [20]. As mentioned, all midwifery schools in Iran have a single educational curriculum. However, in the implementation of this curriculum and in the clinical evaluations of undergraduate midwifery students, we face challenges regarding the practical concepts of midwifery clinical competence. In many midwifery schools, skill is considered as equivalent to competence, especially in clinical assessments, however based on available reliable sources, the course of midwifery has a holistic and women-centered nature [21] and therefore, clinical competence should be considered equivalent to a set of (skill- knowledge-attitude) [10]. Therefore, with aim of clarifying clinical competence in midwifery, we conducted this qualitative study in order to solve the ambiguities and executive challenges of the current midwifery and to have prerequisite for designing a psychometric and valid tool to evaluate the clinical competence of Iranian undergraduate midwifery students.

## Methods

The present study was conducted from March 27 to November 6, 2021, with a qualitative approach and conventional content analysis. Conventional content analysis is commonly used for qualitative studies that aim to describe phenomena about which there are limited research studies. Researchers avoid using predefined categories and prefer to extract categories and subcategories from codes. The research setting in this study included two universities of medical sciences (Guilan and Tehran) and 7 teaching hospitals and 2 health centers affiliated with the universities. The target population included undergraduate midwifery students in the fourth to eighth semesters, midwives working in hospitals and health centers, midwifery faculty members, and obstetricians. Twenty-four people participated in the study, including seven fourth- to eight-semester undergraduate students, nine faculty members of midwifery and reproductive health departments, seven midwives working in hospitals or health centers, and one obstetrician. The participants were selected by purposive maximum variation sampling, which continued until data saturation.

The inclusion criteria were willingness to participate in the interview and signing the consent form to participate in the interview session and have it audio recorded. If any of the participants did not wish to continue with the interview process or have it audio recorded, they were excluded from the study. In this study, data saturation was reached after interviewing 20 people, but four more people were interviewed for greater certainty. The participants were assured that they could withdraw from the research at any time and that all their information would be kept confidential. Individual, semi-structured, in-depth interviews were used to collect the data. The interviews were conducted in person and started with questions such as, “What do you know about clinical competence in midwifery students?”, “In your opinion, what are the components or dimensions of clinical competence in midwifery students?”, and “What traits or behavioral signs in midwifery students are indicative of their clinical competence?” and continued with exploratory questions such as “What exactly do you mean?”, “How?”, “Why?”, and “Please explain this more”. The interviews were audio recorded with participants’ consent, and on the same day, each interview was transcribed verbatim and then coded. The next interview followed the analysis of the previous one. Lincoln and Cuba’s evaluative criteria, including credibility, dependability, confirmability, and transferability, were used to determine the rigor of the qualitative data [22]. To verify the accuracy of the data, the extracted codes were returned to the participants for verification or correction. Trustworthiness was obtained through assessments by the research team members and qualified individuals. Data transferability was ensured through a complete presentation of the research method along with examples of participants’ statements to enable the audience to follow the research path.

## Analysis

The content analysis of the interviews was performed according to the steps proposed by Zhang and Wildemuth (2016)[23], which included:

- **Preparation of data (conducting and transcribing the interviews)**: At this point, the recorded interviews were converted into text format. All the interviews were transcribed to reveal a clear model of participants’ thoughts, behaviors, ideas, and experiences.
- **Definition of the unit of analysis**: Each interview text was entered into the qualitative data analysis software as an analysis unit. Before coding, the entire text of the interview was read several times so that the researcher would get fully acquainted with the data. They were then coded by identifying the meaningful units.
- **Development of categories and the coding scheme**: At this stage, a scheme was formed for the development of categories and subcategories. Categories were extracted inductively from the codes. The codes were first grouped in subcategories based on their similarity, and the subcategories were then grouped based on their relationship with each other to form the categories. The categories were organized in such a way that they had internal compatibility and external incompatibility.
- **Testing the coding scheme in a text sample**: For this purpose, the researcher coded a sample of the text to check the coding consistency by two members of the research team. Disagreements over the coding or categorization rules were resolved through discussions between the research team members.
- **Coding the whole text**: After the researcher and the said two members of the research team reached agreement on the coding consistency, a reproducible process was obtained, and the coding process was generalized to the whole text. During the coding process, the researcher continuously monitored the coding to ensure that there was agreement between the extracted codes based on the researcher’s inference and the views of the study participants and the research team.
- **Assessment of the coding consistency**: After coding the whole text, the coding consistency was assessed again. During the analysis process, the researcher checked the coding consistency, including the initial codes, their placement in the subcategories, and the development of the categories, with other individuals, including the two members of the research team and qualitative research specialists.
- **Drawing conclusions from the coded data, and reporting methods and findings**: At this stage, the characteristics and dimensions of the categories and the relationships between them were determined [15]. MAXQDA software (version 18, VERBI Software GmbH, Berlin) was used for the data analysis.

## Results

The participants included 24 people aged 20–56 years, with a mean age of 39.75 years and mean work experience of 13.62 years (the number of years at the university was considered the students’ work experience). The mean interview time was 48 min. Table 1**presents the demographic characteristics of the participants**. The analysis of the data from participants’ experiences led to the development of four categories (ethical and professional function in midwifery, holistic midwifery care, effective interaction, personal and professional development) and 10 subcategories. Table 2**presents the categories and subcategories resulting from the research.**

**Table 1:** Demographic characteristics of the participants

Number of Participant	Field of Study/Level of Education	Job	Work Experience (years)	Interview Duration (min)
1	Reproductive and Sexual Health Specialist (PhD)	Faculty member of the Midwifery and Reproductive Health Department	10	47:00
2	Obstetrician	Private office and hospital	23	24:57
3	Medical Education Specialist (PhD)	Faculty member of the Midwifery and Reproductive Health Department	25	51:53
4	Master of Midwifery Education	Faculty member of the Midwifery and Reproductive Health Department	6	88:00
5	Master of Midwifery Education	Faculty member of the Midwifery and Reproductive Health Department	19	52:46
6	Master of Midwifery Education	Faculty member of the Midwifery and Reproductive Health Department	11	50:13
7	Reproductive and Sexual Health Specialist (PhD)	Faculty member of the Midwifery and Reproductive Health Department	12	47:07
8	Bachelor of Midwifery	Working at a teaching hospital	28	41:21
9	Bachelor of Midwifery	Working at a teaching hospital	29	41:17
10	Bachelor of Midwifery	Working at a teaching hospital	27	24:03
11	Master of Midwifery Education	Working at a teaching hospital	5	42:51
12	Bachelor of Midwifery	Working at a teaching hospital	21	38:17
13	Bachelor of Midwifery	Working at a teaching hospital	29	72:00
14	Bachelor of Midwifery	Working at a teaching hospital	27	31:00
15	Reproductive and Sexual Health Specialist (PhD)	Faculty member of the Midwifery and Reproductive Health Department	18	46:05
16	Reproductive and Sexual Health Specialist (PhD)	Faculty member of the Midwifery and Reproductive Health Department	7	66:57
17	Midwifery Student semester 8	Student	4	56:13
18	Midwifery Student semester 6	Student	3	18:00
19	Midwifery Student semester 8	Student	4	56:34
20	Reproductive and Sexual Health Specialist (PhD)	Faculty member of the Midwifery and Reproductive Health Department	8	55:52
21	Midwifery Student semester 5	Student	2.5	45:32
22	Midwifery Student semester 7	Student	3.5	52:48
23	Midwifery Student semester 5	Student	2.5	56:02
24	Midwifery Student semester 7	Student	3.5	58:51
Mean of Interview Duration :48 min				

**Table 2:** Categories and subcategories resulting from the research

categories	Subcategories
Category 1. Ethical and professional function in midwifery	1–1. Integration of science and practice in midwifery 1–2. Compliance with ethics and regulations in providing midwifery care 1–3. Improving the independent function of midwifery 1–4. Evidence-based practice
Holistic midwifery care: Category 2	2 − 1. Involving women and their families in providing midwifery and reproductive and sexual health services 2–2. Providing education and support to women and their families for promoting reproductive and sexual health
Effective interaction: Category 3	3 − 1. Using communication skills to provide midwifery and reproductive and sexual health services 3 − 2. Effective and coordinated communication with other people providing midwifery and reproductive and sexual health services
Category 4:Personal and professional development	4 − 1. Feeling responsible and committed to learning for yourself and your peers 4 − 2. Accountability in providing midwifery and reproductive and sexual health services

### Category 1. ethical and professional function in midwifery

This category had four subcategories: The integration of science and practice in midwifery, compliance with ethics and regulations in providing midwifery care, improving the independent function of midwifery, and evidence-based practice.

#### Integration of science and practice in midwifery

Integrating theoretical knowledge in clinical practice is one of the most important skills expected of midwifery students. The participants repeatedly pointed out the importance or weakness of this skill in midwifery students. For example, one participant said, “… I think it is very important that they can put theory into practice -all that time and energy and exams and everything is spent on theory lessons, then we see they can’t put their information into practice in the clinic” (Participant No. 4).

Another participant stressed the need for instructor s to pay attention to nurturing this aspect of competence in students: “… In my opinion, students should know what is important and what should be done for each case they encounter. For example, if there is a mother with placenta previa, they should know what to do in its exact order; the instructor shall also hold a clinical conference the next day based on the cases they’ve had in the ward, as this will help the students know how to put what they have studied in theory into practice” (Participant No. 11).

#### Compliance with ethics and regulations in providing midwifery care

The participants discussed midwifery students’ knowledge about legal and professional standards, and considering that the students become somewhat familiar with the ethical codes of the field in the *Midwifery Ethics and Regulations* course, the expectation was that their practice would be in line with what they had learnt in the course. One of the instructors said about the students observing the principles of confidentiality: “… If, for example, she is going to solve her sexual problem, she has to ask her many private questions. Well, will women trust enough to answer? How should they develop this trust? The woman must feel that she is getting support and confidentiality from the student to answer properly! The student must show by her manner and behavior that she is trustworthy and honest” (Participant No. 4).

Regarding the importance of knowing the framework and limits and the description of the legal duties of the midwifery profession, one of the participants said “… I need to know if what I want to do is in my job description or not. Is it my duty or not? I shouldn’t have false self-confidence. Another thing is that … I should also know what I’m obliged legally to not do and not undertake and which I should refer to my superiors so that there won’t be a problem” (Participant No. 19).

Regarding compliance with the rules set for the particular place of internship, one of the participants said: “… The student must know the importance of enforcing the rules; for example, to enter and exit the place and attend the internship setting, she must follow the rules. She must integrate ethics in all her actions” (Participant No. 23).

#### Improving the independent function of midwifery

Independence of action in midwifery students’ function is one of the components of these students’ practical skills. The participants believed that the students’ practical skills were very important. Regarding the behavioral signs manifested by the students in relation to their practical skills, one of the instructors said: “… A student who can handle 85% of the work she has been assigned without any help is skilled in that work in my opinion. The duration it takes for them to carry out that task is also important, as they should not waste a lot of time!” (Participant No. 7).

One midwife said about the characteristics of a skilled student “… Skill is acquired through work, through experience. In my opinion, a skilled person is someone who takes work seriously, is focused on her work. We or her instructor check her work the first few times. When we see that she does it right, we do not come to check all the time anymore, as we have been assured. I should note that we consider some students our colleagues! Because they do such a great job” (Participant No. 14).

Another participant discussed the students’ interest, enthusiasm, perseverance, and independence of action in performing clinical work: “… The first thing I notice is if the student is willing to do something for the woman; when I see that she is sitting in a corner and has no desire to do anything anywhere at all, I don’t count on her skills then. That is, the eager person will eventually learn, even if she doesn’t already know the job. Eagerness to learn is very important! Some students dare to show their independence of action. Although they are properly monitored from a distance so that no problem occurs, I like this courage because it helps them make progress” (Participant No. 12).

#### Evidence-based practice

In many studies, evidence-based practice is an important principle in healthcare delivery. Several participants interestingly made remarks about this subject. One participant said: “… A student should know where to find the answers to her questions and that what she is doing for the woman is based on scientific evidence and is not a matter of taste. She shall try to keep her principles of care scientifically based and know what kind of articles (aside from their reference books) are valid scientific sources and where they can find those” (Participant No. 1).

Another participant, referring specifically to the exact concept of evidence-based practice, said “… To know how they can find the best evidence! Not just look at the textbook or the clinical guide! Shouldn’t the student be looking for other valid references? Shouldn’t she be able to use articles? Can she discern the quality of the article? Does she understand how credible the content is? Can the results of the article be used as a reference? To what extent? Another thing is to pay attention to the triangle of evidence-based practice, which involves combining one’s own experiences with the best evidence and the woman’s preferences. She should be able to establish this connection, know what the woman wants and what is in her best interest and how much facilities are at her disposal” (Participant No. 3).

### Category 2. Holistic midwifery care

This category includes 2 subcategories of involving women and their families in providing midwifery and reproductive and sexual health services, and providing education and support to women and their families for promoting reproductive and sexual health.

#### Involving women and their families in providing midwifery and reproductive and sexual health services

According to some participants, by providing sufficient explanations to the woman and her family, they can be involved in the treatment process, comprising a mutual benefit. One of the participants said: “… When we explain the treatment process well and the woman is well informed in all our actions, she cooperates with us. Women usually accept the words of those that explain things well to them, and they cooperate with the same person more too” (Participant No. 6).

Referring to the facilitators or inhibitors of effective communication, a student said: “… For example, we had a mother who was an inmate and suspected of being HIV positive. I wanted to teach her about breastfeeding. When I went to talk to her, she was so angry; she said, ‘What do you want to teach me? This is my fourth child, I know everything’. I told her, ‘How interesting! You have four children, so you know everything! So, you teach me how you breastfed your babies! …’ Then her attitude changed completely as she sat down and said what she had done and what she knew. I think I made her feel good by gaining her trust, and actually made her participate” (Participant No. 19).

About the students providing education and showing an appropriate behavior toward the women, one participant said: “… We saw a mother who did not cooperate at all during the birth and was scared and screaming, but she calmed down and cooperated after hearing the appropriate words and behaviors of the student. We were thinking about how it was possible to birth her baby! But after some education and information offered by the student, her self-confidence and patience went up and she cooperated with us and had a very smooth birth” (Participant No. 9).

Regarding the involvement of the woman’s family and companions, one participant said: “… I think it is better for the woman’s company to accompany the woman during the consultation session because they can hear the recommendations and remind the woman of them later to make things smoother and … Yes, it’s important to pay attention to the woman’s family and respect and care for them” (Participant No. 24).

#### Providing education and support to women and their families for promoting reproductive and sexual health

Patient education was one of the most important principles discussed by all the participants in the first minutes of their interviews. In their opinion, proper education empowers women, raises their awareness, and increases their participation in treatment and is one of the important principles of communication between midwifery students and women.

One participant said “… Patient education is also an important part of a student’s skill set. Education is a part of all our work. The student must also get feedback from the woman she has educated” (Participant No. 7).

Another participant’s view on the value of students’ actions to empower women was “… In my opinion, the students should educate women in anything they think can improve their life. For example, if she has come for family planning, the students should ask if they have any other problems to help them with. Like, tell her to live a healthier lifestyle in terms of nutrition, exercise, etc.” (Participant No. 5).

Regarding the efforts by midwifery students to empower women, one participant said “… In general, she shall try to make that woman a more successful member of the society, to empower her by educating and informing her” (Participant No. 3).

### Category 3. Effective interaction

This category contains two subcategories: Using communication skills to provide midwifery and reproductive health services, and effective and coordinated communication with other providers of midwifery and reproductive health services.

#### Using communication skills to provide midwifery and reproductive and sexual health services

Discussions on how to treat and communicate with the women took up most of the interview time. All the participants mentioned this issue and its sensitivity and importance at the beginning of the interviews. Regarding the expression of affection and empathy toward the women, one participant said: “…I say that affection is necessary even before skillfulness. In any difficult situation, if the client receives just the slightest degree of affection, that connection will be established!” (Participant No. 13).

Another participant made the following statement “… I would like to see the student care about the woman, a woman who may even be economically poor or have a low level of education, but the student should not discriminate between the women. That is, for a woman with a low IQ, she shall do the same and show the same attention as she does for an intelligent and well-informed woman, and this unconsciously affects the woman, because it is important for them to be cared for” (Participant No. 9).

Regarding respect for the beliefs and spiritual and religious practices of women, one participant said “… For example, a mother practices religion and would like to recite the Quran. The student must respect her wishes. Or, for example, she likes to carry some prayer in her pocket. The student shall tell her, ‘Yes, this prayer or ritual is a very positive point for you and will help you endure the pain or get well sooner’” (Participant No. 1).

Regarding the importance of the students’ professional behavior with the client, one of the participants stated: “… I expect my students to have a generally professional behavior; that is, they shall not become so intimate with the client that they fail to do the main and important tasks. Being professional means that both she and her client know exactly what has to be done, because the woman doesn’t have a great deal of information! Many times, the woman does not know what decision to make. The student must inform the woman to some extent so that she can make a decision” (Participant No. 16).

Regarding the importance of the students’ provision of humanitarian care, one participant said “… Communicating with the woman is a very important issue for me. The woman should be treated in such a way that she feels comfortable. The student is not here just to learn some tasks and leave! She is not supposed to have an instrumental behavior toward the woman! The student must support the woman. The student should talk appropriately to the women, calm them down, and explain what she is doing for them. Although the student has indeed come to learn to do tasks, communication with the client is also an important skill that must be learned” (Participant No. 8).

Regarding respecting and honoring the clients and their cultural characteristics, one participant said “… For example, our students should have a short friendly conversation with the women before taking a history, and understand that cultures are different. They shall do the examinations with prior permission and with respect and gentleness, be careful when asking questions, as some questions might be taboo for the woman and make her upset or anxious” (Participant No. 15).

#### Effective and coordinated communication with other people providing midwifery and reproductive and sexual health services

All the participants noted the importance of teamwork skills and effective cooperation with the instructor, friends, and staff as an important competence of a midwifery student and expressed their expectations in this regard as follows.

“… Teamwork in midwifery is very important. I think the student’s ability to accept criticism is an important item in this area; of course communication issues depend on individual characteristics to some extent” (Participant No. 6).

“… Communicating with a colleague is good for all of us if it is right. Maybe we all spend like 40% of our time during the day at work. So, if we can communicate properly with our colleagues and create a good environment, it is both in our interest and the other women’s. Communicating with a colleague creates an atmosphere conducive to activity, collaboration, and helpfulness toward the women” (Participant No. 3).

“… Empathy means that a midwife or midwifery student can put herself in the shoes of her colleague or the client at any given moment. That means to understand them at that moment. And where it is necessary, she should support and defend her colleague even when her colleague is not present. And she should try to control her behavior and not act violently. And in the face of violence from others, she should calmly control her own reactions” (Participant No. 21).

“… The student must know what she is allowed to do and what she is not allowed to do. For example, I’ve seen some students who, even if unknowingly, like to make decisions about the woman. They should know what limits a midwife or student have in making decisions and delivering services, and should respect other people. If they want to question the decisions of others, I mean, the midwifery staff’s or physicians’, and have useless discussions with them, it is not right at all!” (Participant No. 11).

“… I always tell students to improve their public relations with the midwifery staff. A student with good public relations is always more successful. I always tell them, ‘Come forward yourself … Volunteer to do the work … First of all, whenever you enter the ward, come and say hello and greet them’. When I see that a student cares about me as a midwife, I also care about her and trust her. I tell myself that this student likes to participate. And when there is a task, for example, finding a vein, I call her to come and do it and to learn. A student with good public relations is successful. Why? Because others leave things to her and she learns in that way” (Participant No. 10).

### Category 4. Personal and professional development

This category consists of the two subcategories of feeling responsible and committed to learning for oneself and for peers and accountability in providing midwifery and reproductive and sexual health services.

#### Feeling responsible and committed to learning for yourself and your peers

One of the interesting points that some participants mentioned was the ability of midwifery students to teach their peers and commitment to continuous learning. Regarding the value of peer education, one participant said “… I saw this positive behavior of teaching each other in the students and I would like for it to spread. For example, with the aim of promoting peer education among the students, I divide the students into pairs to take medical histories together in the first days of the internship. I put a strong student along a weak student so that the weak one can learn indirectly. Actually, peers teach each other better than others do. The comfort that a student feels next to her friend may be much more than that experienced next to me” (Participant No. 7).

Regarding recognizing weaknesses and striving for continuous learning, one participant said “… One should always be able to recognize one’s weaknesses and eliminate them. I expect the students to have this ability as well. For example, she should come and say, ‘I’m not good in finding veins; can I learn from you?’ Their request to learn, the knowledge that they do not know, is valuable to me” (Participant No. 14).

#### Accountability in providing midwifery and reproductive and sexual health services

Accountability was one of the characteristics that were regarded with great importance in the domains of clinical competence among midwifery students. Accountability was in fact considered a principle and the main condition for performing clinical midwifery practices. A participant said: “… I think it is very important for the student to feel accountable; for example, when I deliver a baby, I call from home many times and ask how the mother is. Following up on the woman shows that you both like to do an excellent job and care about the woman; that is, you feel responsible for your client” (Participant No. 9).

“… The sense of responsibility is also an important item. The sense of responsibility is an important part of ethics in midwifery. For example, feeling responsible for educating the mother. This is what I always consider in the evaluation of students, and this will definitely be noticed by the instructor” (Participant No. 13).

One of the midwives said about accepting one’s role as a responsible caregiver: “… I think it is important that everyone knows what their job responsibilities are! The student should know that although she is a student, she is still responsible for that women. If they see an urgent or necessary task, they should attend to it immediately. They should not stay back, but should come forward and do some work” (Participant No. 10).

## Discussion

This was the first qualitative study in Iran examining the domains of clinical competence in undergraduate midwifery students. Based on our findings, the domains of clinical competence in undergraduate midwifery students included four main categories: (1) Ethical and professional function in midwifery; (2) Holistic midwifery care; (3) Effective interaction; (4) Personal and professional development.

Based on the findings of this study, the combination of science and practice in midwifery is an important factor in the correct clinical practice of a midwifery student. Acquiring the ability to integrate theory and practice during clinical midwifery education is essential and has been mentioned in many studies and student experiences about internships [24].

According to studies, the student should be able to integrate knowledge, skills, and attitudes in different clinical situations. A holistic approach that considers the combination of knowledge, skills, and attitudes in midwifery education also facilitates the student assessment process [25].

The importance of the correct application of theoretical information learned in the clinic was expressed in the statements of most of the participants. Accordingly, when a student is actively involved in clinical discussions and rounds, gives correct answers to scientific questions, or can answer the clients’ questions, she can be said to possess appropriate scientific information. Documents published by the Nursing and Midwifery Board of Ireland have emphasized that safe and effective clinical function requires a sound foundation of theoretical knowledge that helps perform clinical practices correctly and improves the students’ scientific mastery of their clinical practice[26].

Also, based on the results of this study, when students critically evaluate and reflect the sources and evidence to find low-risk and high-quality solutions, and when they demonstrate correct clinical practice and judgment based on evidence, they can then be said to have properly demonstrated the integration of science and practice in midwifery. According to documents published by the Nursing and Midwifery Board of Ireland, critical thinking, problem-solving, and decision-making skills are essential capabilities for the art and science of midwifery [26].

Regarding respect for ethical and professional rights in the provision of midwifery care, gaining the women’s trust as a professional person was a key point, which was described with behavioral signs such as getting the women’s permission before any examinations or before touching their body for procedures such as vaginal and breast examinations, taking a history by asking questions politely, paying attention to the women’s cultural background and mental taboos, instilling a sense of confidentiality and professionalism in providing services to the women, and maintaining the women’s privacy and dignity. Other studies have reported that women want to be informed about the interventions and examinations performed on them and consider this behavior a prerequisite of respectful care [27, 28]. Women believe that midwives should ask for their permission before performing procedures such as vaginal or breast examinations [29–32]. Similarly, several studies have identified the importance of informed consent as part of respectful maternal care [33, 34]. The concept of moral competence has been defined as having moral awareness, moral judgment skills, a strong personality, and a desire to do good deeds [35]. One of the roles of midwifery education is to enable the students to acquire these skills. According to other studies, midwifery students need to develop critical thinking skills, reflective thinking empathy, self-awareness, knowledge of ethical codes, communication skills, and high-quality teamwork skills in order to achieve moral competence [36, 37].

These competences are also mentioned in the international standards set forward by the International Confederation of Midwives (ICM) and the American College of Nurse-Midwives (ACNM) [16, 38].

Also, in the field of professional ethics, it was important for the participants of our study that midwifery students be aware of the limits of their professional responsibility and not expect to learn or practice functions or skills outside their area of ​​responsibility as an independent midwife in the future. Other professional, legal, and ethical behaviors included the implementation of workplace health and safety guidelines, the implementation of infection control procedures, clean and tidy appearance and clothing with respect to the professional dress code, and the proper completion of legal documentation in the women’s files.

Promoting independent midwifery function is one of the main goals of students’ presence in clinical settings. According to the results of this study, efforts to improve clinical performance in midwifery students have obvious behavioral symptoms that are highly regarded by educators and clinical staff. According to a study by Fullerton et al. (2011), confidence in midwifery care is essential because the profession requires the ability to work independently with a scientific approach and a sense of professional responsibility. Self-confidence has been described as “the ability to do work successfully and efficiently” [39]. One of the results reported in the study by Back et al. (2020) in Sweden also showed the importance of self-confidence in midwifery students in clinical settings, where physicians are increasingly dominant [24]. In our study, improving the independent function of midwifery and boosting the students’ self-confidence in clinical settings, where obstetrician residents are the dominant force, was considered a challenging issue for most participants.

In the present study, evidence-based practice was one of the areas of ​​clinical competence in midwifery students. The participants in our study expected midwifery students to understand the value of the latest credible scientific evidence in the clinic and be able to seek scientific sources, especially while at the patient’s bedside, to provide appropriate services to them and ultimately adopt low-risk and effective practices. Evidence-based practice is a patient-centered, holistic, and problem-solving-oriented approach to healthcare delivery that combines the best available evidence with healthcare providers’ experiences and patients’ preferences to make the best decision for the patient [40]. According to the Nursing and Midwifery Board of Ireland, in the complex and unstable clinical environment, it is imperative to use the best available evidence in clinical practice. We can therefore expect that all healthcare providers throughout the world be well acquainted with the importance of evidence-based practice, which must also be applied in clinical practice and taught in educational programs; however, this is not necessarily the case all the time. Implementing evidence-based practice in healthcare provision, including nursing and midwifery, remains a major global challenge [41]. A study by Cleary et al. (2021) concluded that although physicians, instructors, and students believe that evidence-based practice is the best standard of care for patients, they still express a lack of confidence in their ability to perform or teach it. They have attested to their poor performance of evidence-based practice in the clinic, stating that the culture of evidence-based performance is still weak in many cases. According to them, today’s students are the nurses and midwives of tomorrow and they must be educated and experienced in evidence-based practice [42]. In the present study, midwifery instructors and staff and even students were concerned about the poor development of clinical competence in students in this area despite attesting to the importance of evidence-based practice. The participants stated that evidence-based practice skills do not develop even until graduation.

According to the results of our study, one of the important goals of midwifery education programs is to involve women and their families in providing reproductive and sexual health services in the form of a commitment to the provision of women-centered care. Women-centered care is a genuine philosophy for midwifery that forms a participatory and interactive relationship between women and midwives by recognizing and combining the social, emotional, physical, spiritual, and cultural needs of women, along with the knowledge and expertise of the midwifery profession [13, 14, 43–48]. In woman-centered care, the needs and context of women’s health are defined by the women themselves [49–51]. Women-centered care is associated with positive birth outcomes and positive maternal experiences [52]. In current midwifery practices, there is a strong emphasis on supporting and respecting the values, preferences, choices, and concerns of every woman during the period in which she receives midwifery care [49, 50]. In one study, Dutch midwives reported that providing women-centered care in daily clinical practice was difficult and challenging [53, 54]. These findings are in line with the results of our study. The midwives and midwifery instructors participating in our study attributed their own and the midwifery students’ weaknesses in this skill to the environmental conditions and the predominant role of obstetricians and obstetrician residents in clinical settings. Other studies suggest that the basics of learning, understanding, and commitment to women-centered care should be included in midwifery education programs [49] and strengthened and enriched by active participation in clinical work [55–58].

According to the results of our research, the midwifery student should be educated in such a way that she can plan a care program with the participation of women and acquaint them with their health rights as a woman. The midwifery student should also be able to communicate respectfully with the women’s company and relatives and provide honest and intelligible answers to their questions about healthcare processes. In other studies, midwives have demonstrated a strong belief in the women’s right to participate in and make decisions [33, 59, 60]. Based on the evidence, healthcare providers value the interaction and active participation of the women’s families in their care [61, 62].

Based on the results of our research, regarding educating and supporting women and their families to improve their reproductive and sexual health, the participants believed that a qualified midwifery student always tries to support women, mothers and their families, provide counseling and information on reproductive and sexual health tailored to their individual circumstances and health promotion needs, and receive feedback after each education to ensure they have properly understood every item. The participants also noted the importance of providing counseling to the women and their families and stressed that the future midwife should have the characteristics of a capable counselor. According to the participants in our study, patient education is an important component of the clinical competence of a midwifery student and an important aspect of respectful maternal care. Nevertheless, there are weaknesses in cultivating this skill among the students that must be corrected. According to studies, patient education is a dynamic and continuous process that improves the patients’ knowledge, skills, and motivation to maintain and promote their health, but usually, patient education efforts are of a low quality and do not result in a very successful transfer of the required knowledge and skills to the patients [63, 64].

In Iranian hospitals, patient education is associated with many challenges and could benefit from getting incorporated into the students’ curriculum and evaluated [65].

According to the participants in our study, the skill of communication with women is very important, especially in the midwifery profession, because an important part of midwifery duties is to provide care during labor, a period in which women need to be respectfully and empathically supported and cared for due to their special circumstances and labor pain. Sufficient patience in dealing with the women, appropriate verbal and non-verbal communication with them, understanding cultural diversity, and establishing purposive interaction with the women constitute important communication skills expected of a midwifery student.

According to many studies, both women and healthcare providers around the world have emphasized the importance of effective communication as a key component of respectful maternal care. Women have always appreciated receiving verbal encouragement during labor and birth and have highly valued the emotional support they receive from midwives during this critical time [28, 32, 59, 66–75].

Based on the available evidence, healthcare providers consider talking and listening to women, a vital part of humanitarian care and have always attributed significant value to showing empathetic behaviors toward women [27, 76].

In many studies, healthcare providers have emphasized the importance of showing respect for women’s different cultures, values, and beliefs [28, 34, 59, 67]. For example, Muslim women in many countries like only for female healthcare providers to serve them before and during labor, and this demand must be respected [59, 77, 78]. For healthcare providers, the term cultural competence means having the attitude, knowledge, and skills required to provide high-quality care to ethnic groups with different cultures. Studies have shown that teaching the principles of cultural competence to healthcare providers can improve this aspect of healthcare providers’ competence in general [79–81].

Fair et al. (2021) concluded that education related to cultural competence has generally been welcomed by midwives. They felt that this education would affect the quality of the care they provide to immigrant women who have recently moved to that country [82].

Studies have recommended that faculty members should receive further support to teach the subject of cultural safety in their undergraduate courses. In addition, cultural safety lessons should be incorporated into the empowerment programs targeting health science instructors [83]. According to the results of our study, midwifery students should be able to observe justice in their performance and avoid a biased performance of their duties. Another crucial point is to understand and support the women when they are experiencing a psychological crisis or stress. Also, gaining the women’s trust as a professional healthcare provider and respecting the beliefs and spiritual and religious practices of the women are other points that can be expected of midwifery students in their practice.

These points are also mentioned in a qualitative review study by Shakibazadeh et al. (2018). Promoting respectful maternal care is increasingly recognized as a vital element contributing to the quality of midwifery care. All women need and deserve respectful care. Respectful maternal care should be considered a vital component of providing good quality care to mothers and infants in health systems. Nonetheless, university curricula focus primarily on medical care rather than the humanitarian aspects of care; therefore, healthcare providers have always supported the inclusion of lessons on respectful maternal care in midwifery education. It has also been stated that women prefer receiving services from healthcare providers who are kind, calm, tactful, warm-hearted, smiling, and supportive and spend more time attending to them [5].

Insisting that midwifery students should possess characteristics such as empathy and show love and affection and use beautiful and encouraging words toward parturient women along with the presence of powerful role models such as experienced educators and midwives were repeatedly discussed by our study participants.

According to many participants in our study, effective and coordinated communication with other people providing reproductive and sexual health services has an important role in students’ clinical learning. Therefore, weaknesses in effective communication with healthcare providers in clinical settings can have a significant impact on the quality of learning clinical skills in midwifery students. According to the participants of our study, positive interaction between midwifery students and staff can be effective in creating learning opportunities for students. Instructors and healthcare staff expect a midwifery student to accept criticism and be compatible with other healthcare providers in the clinical setting. A very important point mentioned was the importance of active student participation in teamwork. The participants stated that despite the importance of teamwork skills in a midwife’s future career, students lack this skill. Clinical instructors were expected to clarify the importance of teamwork skills to students in order to strengthen this skill in them. Another point was the emphasis placed on the students’ ability to deal with violent behaviors in the clinical workplace. The expectation was that the student should be able to react appropriately in these situations, also be able to cope with and resolve personal stresses and maintain composure in the clinical workplace. Also, appropriate professional behavior with the healthcare team members and respect for their opinions was mentioned as an important factor in creating effective interactions between the students and clinical staff and thus promoting student learning.

According to Patterson et al. (2021), the skill and safety of midwifery practice in addition to practical and interpersonal skills depends on the ability to successfully participate in interprofessional challenges and dialogues and contributes to maternal and neonatal health. They suggested including opportunities to help learn these skills in practical midwifery curricula. The use of simulated education methods can also help build confidence and improve inter-professional collaboration skills in midwifery students [84].

Bullying and aggression in the workplace indicate physical, verbal, social, or psychological abuse by an individual or a group of people in the workplace [85]. This behavior is a cause for concern because it can cause the students to withdraw from the educational environment before completing their studies [86]. Bullying and aggression by staff or patients can disrupt the students’ learning experiences and prevent them from socializing professionally, thereby preventing improvements in the healthcare provided to the patients [87, 88].

Bullying is common in clinical settings. Hospital staff and midwifery instructors are the main sources of bullying in clinical settings. Patients and their company are also sometimes involved in this issue [89].

According to a study by Birks et al. (2017), bullying tendencies in the workplace can be controlled by learning certain skills. For example, a series of education sessions can be effective in promoting resilience and strengthening emotional intelligence in the face of negative situations [90]. Online resources, interactive modules with realistic scenarios, and practicing role-play of the skills prepares students to deal with bullying and aggression in clinical settings [91].

According to the participants of our study, there are clear behavioral signs in students regarding feeling responsible and committed to learning themselves and helping their peers learn. They stated that some students had never acted selfishly and were sensitive to their peers’ learning too, took turns to take advantage of the educational opportunities offered, especially for the rarer cases, and monitored and guided their peers to address any shortcomings in clinical skills. The participants considered this behavior commendable and believed that such behaviors should be a role model for other students.

We believe that the commitment to learning for oneself and one’s colleagues at the university will establish this behavior in the student’s future career. Therefore, these behaviors should be reinforced and encouraged in students by educators. The value of commitment to learning for one and helping others learn has also been reported in other studies [21, 92].The results of a qualitative study in Sweden showed that learning from peers has positive consequences. In this type of learning, students share their skills, experiences, and knowledge equally and take responsibility for each other’s work during their internships. Students share their ideas, thoughts, and knowledge and gain new perspectives on clinical learning at equal levels, and with the help of each other, they can function independently without the intervention of an instructor .Active learning takes place in peer learning models, in which the student is in the spotlight and the instructor has a facilitating role [93]. Increased self-confidence [94], self-efficacy [95], reduced anxiety, safer learning environment [96], greater independence of action, and strengthened teamwork skills [94] are other benefits of peer learning.

The participants also noted the importance of behaviors that demonstrated accountability in providing reproductive and sexual health services. Based on the results of our study, this can be part of the personal and professional development of the midwifery student. Students were expected to carefully follow the assigned tasks, be responsible and accountable in their professional practices, and be sure to ask for help when they did not have sufficient clinical skills and knowledge. The participants considered such a student trustworthy and demanded the presence of such students in clinical wards. The importance of accountability in providing midwifery services has also been emphasized in ICM [16].

## Conclusion

The domains of clinical competence in midwifery derived from our study and their importance are consistent with those concluded in many similar studies. The findings of the present study showed that clinical competence in midwifery students includes different domains and corresponds to the general definitions of clinical competence overall, as it includes skills, knowledge, and attitude in clinical practice. To have qualified working midwives in the future, it is necessary to cultivate the main domains of competence during their student years. Instructors and education managers are advised to pay further attention to the domains of clinical competence in midwifery students in accordance with the conditions of their community health systems and to help improve the necessary competences through formative clinical evaluations to guide midwifery students. The results of this study can be used as a basis for designing and the psychometric assessment of a clinical competence assessment tool for undergraduate midwifery students.

## Data Availability

The datasets used and analysed during the current study available by the corresponding author on reasonable request.

## Declarations

### Limitations of this study

The main limitations of this study is related to the nature of all qualitative studies, that is, the lack of generalizability. Of course, we tried to minimize this weakness by sampling with purposive maximum variation sampling.

### Strengths of this study

1-The importance of this study is that no comprehensive study has been conducted on the dimensions of clinical competence in midwifery students in Iran.

2-Providing input with regard to the long experience of authors as a member of the academic staff and clinical instructor of undergraduate midwifery and nursing students.

3-Existence of participants with clinical experience and interest and long record of work in this field, most of whom are concerned about the progress of midwifery education.
